# Genome-Wide Association Study of Ureide Concentration in Diverse Maturity Group IV Soybean [*Glycine max* (L.) Merr.] Accessions

**DOI:** 10.1534/g3.115.021774

**Published:** 2015-09-14

**Authors:** Jeffery D. Ray, Arun Prabhu Dhanapal, Shardendu K. Singh, Valerio Hoyos-Villegas, James R. Smith, Larry C. Purcell, C. Andy King, Debbie Boykin, Perry B. Cregan, Qijian Song, Felix B. Fritschi

**Affiliations:** * Crop Genetics Research Unit, USDA-ARS, Stoneville, Mississippi 38776; † Division of Plant Sciences, University of Missouri, Columbia, Missouri 65211; ‡ Crop Systems and Global Change Lab, USDA-ARS, Beltsville, Maryland 20705; § Department of Plant, Soil and Microbial Sciences, Michigan State University, East Lansing, Michigan 48824; ** Department of Crop, Soil, and Environmental Sciences, University of Arkansas, Fayetteville, Arkansas 72704; †† Southeast Area Statistics, USDA-ARS, Stoneville, Mississippi 38776; ‡‡ Soybean Genomics and Improvement Lab, USDA-ARS, Beltsville, Maryland 20705

**Keywords:** ureide, drought tolerance, soybean, GWAS

## Abstract

Ureides are the N-rich products of N-fixation that are transported from soybean nodules to the shoot. Ureides are known to accumulate in leaves in response to water-deficit stress, and this has been used to identify genotypes with reduced N-fixation sensitivity to drought. Our objectives in this research were to determine shoot ureide concentrations in 374 Maturity Group IV soybean accessions and to identify genomic regions associated with shoot ureide concentration. The accessions were grown at two locations (Columbia, MO, and Stuttgart, AR) in 2 yr (2009 and 2010) and characterized for ureide concentration at beginning flowering to full bloom. Average shoot ureide concentrations across all four environments (two locations and two years) and 374 accessions ranged from 12.4 to 33.1 µmol g^−1^ and were comparable to previously reported values. SNP–ureide associations within and across the four environments were assessed using 33,957 SNPs with a MAF ≥0.03. In total, 53 putative loci on 18 chromosomes were identified as associated with ureide concentration. Two of the putative loci were located near previously reported QTL associated with ureide concentration and 30 loci were located near genes associated with ureide metabolism. The remaining putative loci were not near chromosomal regions previously associated with shoot ureide concentration and may mark new genes involved in ureide metabolism. Ultimately, confirmation of these putative loci will provide new sources of variation for use in soybean breeding programs.

A genome-wide association study (GWAS) is an alternative to QTL mapping of biparental populations, is widely used in human genetics, and is increasingly used in plant studies. The GWAS approach typically requires a high density of single-nucleotide polymorphisms (SNP) across the genome of a large number of individuals, as well as phenotyping of all individuals in the study. Significant statistical associations are then determined between SNP alleles and the trait phenotype. Soybean traits analyzed by GWAS include mineral deficiency (iron and aluminum), seed traits (protein, oil, size, and shape), disease resistance (cyst nematode and soybean mosaic virus), environmental responses (temperature, drought, salt, and photoperiod), as well as seed yield and yield components (reviewed by Deshmukh *et al.* 2014).

Not included in the review by Deshmukh *et al.* (2014) was a report that identified genomic regions related to P efficiency (Zhang *et al.* 2014), sudden death syndrome (SDS) (Wen *et al.* 2014), and more recently published reports identifying genomic regions related to carbon isotope discrimination (Dhanapal *et al.* 2015a) and N concentration, N derived from atmosphere, and C:N ratio (Dhanapal *et al.* 2015b). Further, three qualitative traits (flower, hilum, and pubescence color) and three quantitative traits (maturity, plant height, and seed weight) were recently analyzed by GWAS (Sonah *et al.* 2014). It is likely that the number of soybean GWAS reports will substantially increase with the recent release of the SoySNP50K (Illumina iSelect SNP BeadChip) data for more than 19,000 accessions of the USDA-ARS Soybean Germplasm collection (Song *et al.* 2013).

Within statistical parameters, GWAS can identify genomic regions (SNP alleles) associated with the traits of interest. However, it is expected that these regions will require independent validation. Nonetheless, reducing the genome to fewer regions of interest for further study is a significant accomplishment. In a recent GWAS study on soybean protein and oil (Hwang *et al.* 2014), most previously reported quantitative trait loci (QTL) for these traits were identified and the genomic regions in which the QTL were identified was narrowed. Results such as these provide a measure of confidence in GWAS findings in soybean and the potential of GWAS as a research tool to identify genomic loci for traits of interest.

Ureides (allantoin and allantoate) are the N-rich products of N-fixation that are transported from soybean nodules to the shoot. Considerable research has focused on the metabolism of ureides, especially with regard to drought. Ureides have long been thought to play a role in the sensitivity of N-fixation to drought that may involve a feedback inhibition resulting from accumulation of ureides in leaves and nodules during water-deficit stress (Sinclair and Serraj 1995; Serraj and Sinclair 1996; de Silva *et al.* 1996; Gordon *et al.* 1997; Purcell *et al.* 1998; Serraj *et al.* 1999, 2001; Vadez *et al.* 2000; King and Purcell 2005; Ladrera *et al.* 2007). Coleto *et al.* (2014) concluded that although ureide accumulation was a general stress-related response and not the cause or signal of N-fixation inhibition in common bean (*Phaseolus vulgaris* L.), there was a greater concentration of ureides in shoots of drought-sensitive genotypes than drought-tolerant genotypes, which is similar to results for soybean (King and Purcell 2005; King *et al.* 2014). Watanabe *et al.* (2014) suggested a possible regulatory action for allantoin in which it influences abscisic acid production, thereby affecting stress tolerance.

Although the exact role of shoot ureide accumulation in the downregulation of N-fixation has not been elucidated, a preponderance of evidence indicates that ureides may be useful in identifying genotypes that are able to continue N-fixation at relatively low soil moisture content (Sinclair *et al.* 2000; King and Purcell 2005; King *et al.* 2014). For example, Sinclair *et al.* (2000) used a low petiole ureide concentration as a preliminary screen to identify genotypes that might be more drought-tolerant from approximately 3000 soybean accessions. Genotypes with the lowest 10% petiole ureide concentration were selected for more selective screens, which ultimately identified eight accessions with drought-tolerant N-fixation. Our objectives in this research were to measure shoot ureide concentration in a large group of soybean accessions [374 Maturity Group (MG) IV accessions] and conduct GWAS to identify genomic regions associated with shoot ureide concentration.

## Materials and Methods

### Field experiments

Field experiments were conducted at two locations (Columbia, Missouri and Stuttgart, Arkansas) over 2 yr (2009 and 2010). In Columbia, the experiments were conducted at the Bradford Research and Extension Center (38° 53′N, 92° 12′ W) and in Stuttgart, they were conducted at the Rice Research Experiment Station (34° 30′ N, 91° 33′ W). For the purpose of analysis and discussion, each year and location was treated as a separate environment and designated as C09, C10, S09, and S10 for Columbia and Stuttgart in 2009 and 2010. Experimental details were described by Dhanapal *et al.* (2015a). The soil texture at both locations was a silt loam [in Columbia, a Mexico silt loam (fine, smectitic, mesic Aeric Vertic Epiaqualf) and in Stuttgart, a Crowley silt loam (fine, montmorillonitic, thermic Typic Albaqualfs)] and the fields were tilled prior to sowing. In both years, sowing occurred earlier in Columbia (May 23, 2009 and May 27, 2010) than in Stuttgart (June 2, 2009 and June 10, 2010). In both years, four-row plots were used in Columbia and single-row plots were used in Stuttgart. Fertilization was based on soil tests and followed the recommendations of the University of Missouri (http://aes.missouri.edu/pfcs/soiltest.pdf) and the University of Arkansas (http://www.uaex.edu/publications/pdf/mp197/chapter5.pdf). Furrow irrigation was applied to experiments S09 and S10 as needed and experiments C09 and C10 were grown without irrigation (*i.e.*, rainfed).

### Experimental design

As reported by Dhanapal *et al.* (2015a,b), 385 soybean [*Glycine max* (L.) Merr.] MG IV accessions were sown in a randomized complete block design with three replications in all four environments (two locations and 2 yr; C09, C10, S09, S10). The accessions evaluated were obtained from the USDA-ARS Germplasm collection based on GRIN (Germplasm Resources Information Network, www.ars-grin.gov) data and with the assistance of the collection curator, Dr. Randall Nelson. The selected accessions were all MG IV genotypes with seed yield >1.7 Mg ha^−1^ and good agronomic traits (height, lodging, shattering, etc.). Because SNP data on the germplasm collection were not yet available at the time when entries were selected, genetic diversity was estimated by considering country and province of origin in proportion to the number of entries from that source in the germplasm collection. For the ureide trait described in this analysis, data on 374 of the 385 accessions are reported and analyzed.

### Ureide sampling and analysis

The above-ground portions of five plants chosen at random from each plot were harvested between beginning bloom (R1) to full bloom (R2) [stages according to Fehr *et al.* 1971)]. In Columbia, sampling took place 53 d after planting (DAP) in both years, and in Stuttgart they were conducted 50 DAP in 2009 and 61 DAP in 2010. Harvested plants were dried in an oven at 60° until completely dry. The five-plant samples were then ground in a Wiley Mill (Thomas Model 4 Wiley Mill; Thomas Scientific, NJ, USA) to pass through a 2-mm screen, mixed, and then a subsample was ground a second time using a UDY Cyclone sample mill with a 1-mm screen (MODEL 3010-014; UDY Corporation, CO, USA). Ureides were extracted by placing 0.125 g of the ground shoot material in a test tube with 5 ml of 0.2 M NaOH. After placing test tubes in a water bath at 100° for 30 min, a 1-mL aliquot was transferred to a 1.5-mL microfuge tube and centrifuged at 20,000 × *g* for 5 min. Fifty to 100 µL of the supernatant was analyzed for ureides using the colorimetric procedure of Young and Conway (1942).

### Analyses

In each environment (C09, C10, S09, and S10), the experimental design was a randomized complete block. Ureide values were log-transformed to equalize variance among environments. ANOVA was conducted on the log-transformed values within and across environments using a general linear mixed model with environments and accessions treated as random effects. Additionally, accession mean ureide concentrations were obtained using BLUP (best linear unbiased prediction) predictors by Proc GLIMMIX of SAS (ver. 9.3; SAS Institute, Cary, NC). Ureide phenotypic means are shown in Supporting Information, File S1.

SNP data on the 374 accessions evaluated in this study were obtained from the SoySNP50K iSelect SNP BeadChip (Song *et al.* 2013) now curated on SoyBase (www.soybase.org). Brief instructions for obtaining the SNP data are shown in File S2. For the 374 accessions, 33,957 SNPs had a minor allele frequency (MAF) of ≥3%, which was the threshold for inclusion in the analysis reported herein. This threshold was chosen to facilitate the identification of rare genotypes. BLUP predictors of ureide accession means derived for each individual environment, and also across all environments, were used for genome-wide association analysis. Linkage disequilibrium (LD) was calculated using all SNPs with a MAF ≥3% among the 374 soybean accessions and distributed over the 20 soybean chromosomes. Calculation of pairwise LD (r^2^) among SNPs was based on SNPs within a 1-Mb window using PLINK (Purcell *et al.* 2007) software. Separate LD calculations were performed for euchromatic and heterochromatic chromosomal regions.

JMP Genomics 7.1 (SAS Institute, Cary, NC) was used to perform the genome-wide association analysis and to generate covariate matrices to account for population structure (Q-matrix) and genetic relatedness (K-matrix). First, the K-matrix was generated using allele sharing similarity, and from this the Q-matrix with eight dimensions (Dhanapal *et al.* 2015b) was generated using multidimensional scaling (Kruskal and Wish 1978; SAS Institute 2008) to identify grouping patterns. However, the K-matrix used in the genome-wide association analysis was generated using identity-by-descent. Because the Q-matrix is derived from the K-matrix, part of the Q/K relationship is strictly due to this computation. Using both measures provides a more conservative accounting of population structure. The Null Model Likelihood Ratio Test (SAS ver. 9.4; SAS Institute, Cary, NC) indicated that the Q-K model significantly (*P* ≤ 0.005) improved the description of variance between genotypes in all environments as compared to a model without adjustment for genetic relatedness. Both matrices were generated using all 33,957 SNPs with a MAF ≥3%. These matrices were used with the Q-K Mixed model procedure (PROC GLIMMIX) to test for association between ureide concentration and SNP while simultaneously adjusting for population structure and genetic relatedness (Yu *et al.* 2006). The model used fixed effects for SNP and each element in the Q-matrix as a covariate and random effects for each element in the K-matrix as a covariate.

SNP–trait associations were conducted on ureide concentration within individual environments as well as for the average ureide concentration across all four environments. Within environments and for the average across environments, the threshold for declaring a significant association was set to *P* ≤ 0.0001, which is comparable or more stringent than that reported in other soybean GWAS studies (Hao *et al.* 2012; Hwang *et al.* 2014; Mamidi *et al.* 2014; Zhang *et al.* 2015).

To take advantage of the four independent environments utilized in this study, the within-environment results were further analyzed by considering the joint probability for all possible two-environment and three-environment combinations. By definition (Mendenhall and Scheaffer 1973), the joint probability of being wrong both times is the probability of falsely rejecting the test of significant SNP effect in one environment × the probability of falsely rejecting the test of significant SNP effect in the other environment. Joint probabilities were calculated by multiplying the respective *P* values of each SNP in all possible two-environment and three-environment combinations. A multiple testing adjustment (FDR) (Benjamini and Hochberg 1995) at a threshold of *P* ≤ 0.01 was applied across all SNPs and joint probabilities collectively using the “*P*-Value Adjustment” of JMP Genomics v7.1. By-environment probabilities, joint probabilities, and adjusted joint probabilities are shown in File S3. Six SNPs identified in these analyses that showed conflicting results were eliminated as likely false-positive results.

### Data availability

File S1 contains the ureide means of the 374 plant introductions for the four environments. File S2 describes how to obtain the SNP data from www.soybase.org. File S3 contains the by environment probabilities, 2-environment joint probabilities and FDR adjusted 2-environment joint probabilities for all 33,397 SNPs tested for significant associations with ureide content. Table S1 shows the SNP information for all SNPs identified as significantly associated with ureide concentration.

## Results

### Environment and accessions

**Figure 1:**
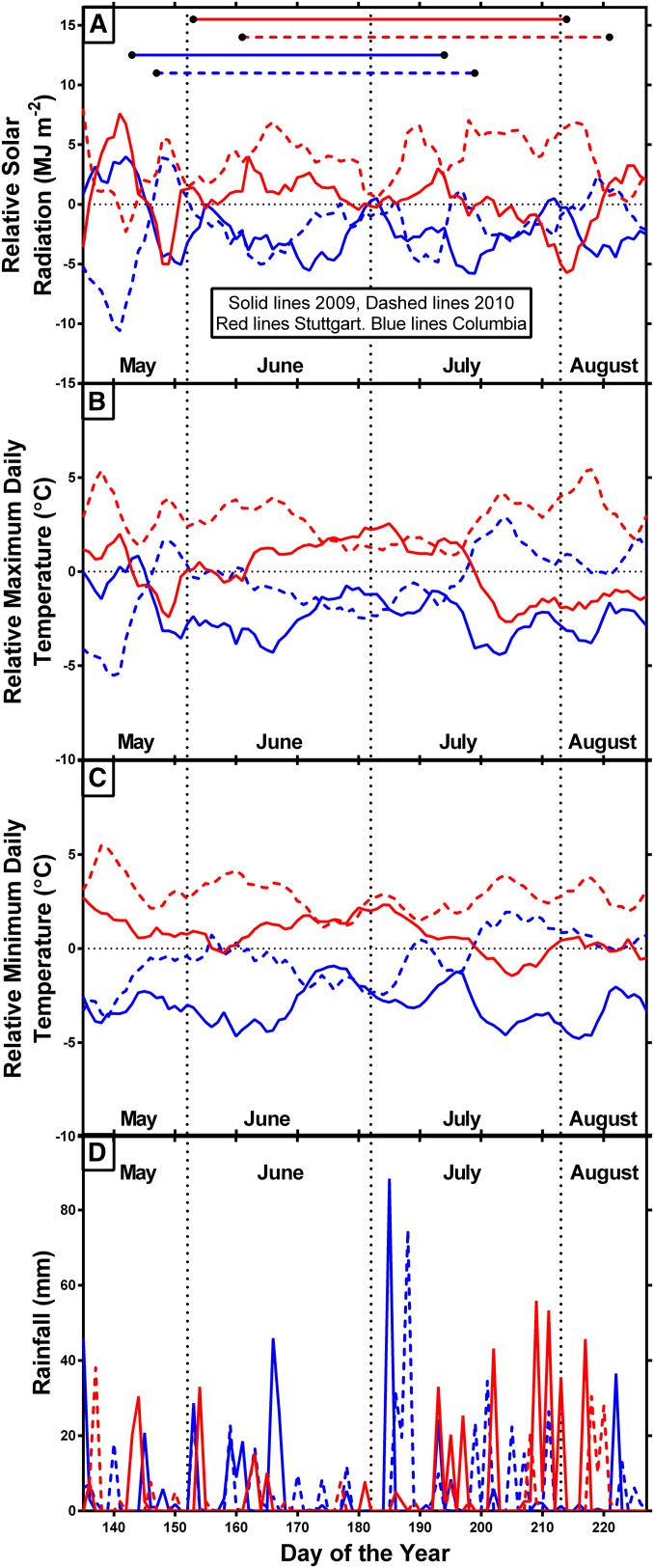
The 7-d running average solar radiation (A), maximum temperature (B), and minimum temperature (C) relative to the average across all four environments (indicated by the zero line). Rainfall is shown as the unadjusted daily rainfall (D). Horizontal lines in (A) indicate the growing period between planting and sampling.

Although environmental conditions were generally more similar between years in Columbia and in Stuttgart than between the locations, considerable variation in environmental conditions was observed among all four environments (Figure 1). Solar radiation generally was greater in Stuttgart than in Columbia for both years and generally greater in 2010 than in 2009 for Stuttgart (Figure 1A). The relative maximum and minimum temperatures (Figure 1, B and C) indicated that Stuttgart in 2010 was the warmest environment and Columbia 2009 was the coolest environment. Overall, Columbia received more rainfall than did Stuttgart (Figure 1D), but the experiments in Stuttgart were supplemented with irrigation as needed (data not shown).

Ureide concentration data were obtained on a total of 374 accessions in each of the four environments. Overall, the accessions represented 11 different national sources and a total of at least 37 different provinces within those countries (Table 1). Based on population structure analyses for these accessions and the SNP data set, Dhanapal *et al.* (2015a,b) previously determined that they could be grouped into eight subpopulations.

**Table 1:** Country of origin and province (if known) of the 374 accessions evaluated for ureide concentration in four environments (two locations and 2 yr)

Country	Province	No. of Accessions	Country	Province	No. of Accessions
China	Anhui	3	Korea	Unknown	4
	Beijing	2	Mexico	Jalisco	1
	Fujian	1	North Korea	Unknown	11
	Gansu	1	Romania	Unknown	1
	Hainan	1	Russia	Krasnodar	1
	Hebei	9		Unknown	1
	Heilongjiang	1	South Korea	Cheju	1
	Henan	2		Cholla Nam	18
	Jiangsu	5		Cholla Puk	17
	Jilin	2		Chungchong Nam	39
	Liaoning	3		Chungchong Puk	13
	Shaanxi	1		Kangwon	29
	Shandong	17		Kyonggi	41
	Shanxi	4		Kyongsang Nam	40
	Sichuan	3		Kyongsang Puk	30
	Unknown	6		Seoul	7
Georgia	Unknown	6		Unknown	9
India	Unknown	1	Taiwan	Unknown	2
Japan	Akita	5			
	Hokkaido	1			
	Hokuriku	1			
	Iwate	1			
	Kanto	13			
	Kinki	1			
	Kyushu	1			
	Miyagi	1			
	Nagano	3			
	Tohoku	10			
	Unknown	4			

Using all SNPs with a MAF ≥0.03 and all 374 genotypes, LD was separately calculated for euchromatic and heterochromatic chromosomal regions. In euchromatic regions the mean LD (r^2^) declined to 0.2 within approximately 185 kbp, which is approximately half that reported by Hwang *et al.* (2014) and Zhang *et al.* (2015) for a similar number of SNPs but with different and fewer soybean accessions. However, it was very similar to that reported by Dhanapal *et al.* (2015a) for the same set of soybean accessions used herein, but with fewer SNPs. As reported by others (Hwang *et al.* 2014; Zhang *et al.* 2015), the LD was very different in euchromatic regions than in heterochromatic regions. In this study, LD in the heterochromatic regions did not decay to half of the maximum value within 1 Mb, which was similar to that reported by Dhanapal *et al.* (2015a).

**Figure 2:**
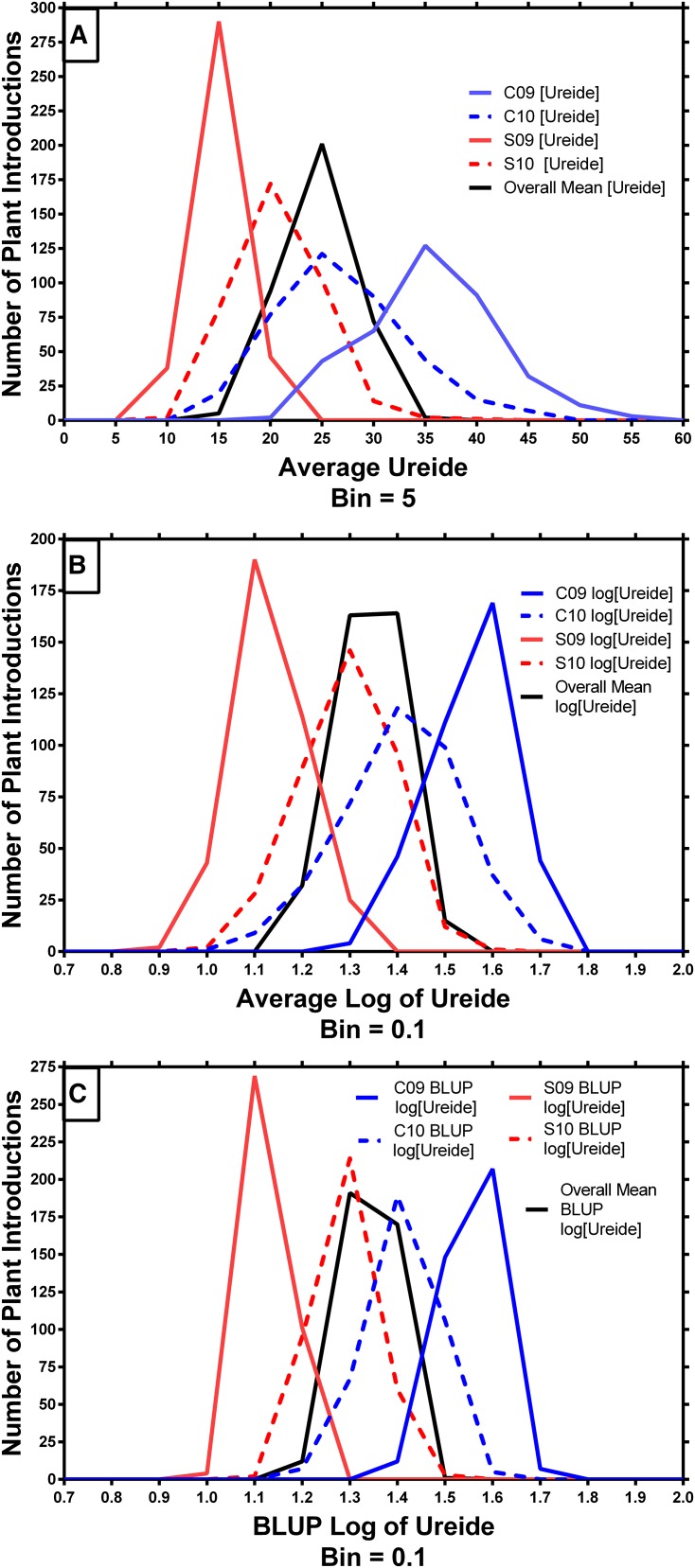
Distribution of the three-replicate mean ureide concentrations across all 374 genotypes evaluated for each of the four environments. The unadjusted means (A), the log-transformed distributions (B), and the distribution of the log-transformed BLUP means (C). SNP–trait associations were conducted on the log-transformed BLUP means.

The average shoot ureide concentrations across all four environments and 374 accessions ranged from 12.4 to 33.1 µmol g^−1^ with a minimum to maximum range from 7.4 to 50.5 µmol g^−1^ (Table 2; Figure 2A). These values were comparable to previously reported values for a set of 96 recombinant inbred lines that ranged from 18.6 to 39.0 µmol g^−1^ across 4 yr experimentation with a minimum to maximum range of 9.8 to 64.0 µmol g^−1^ (Hwang *et al.* 2013). In both years, the average ureide concentration was higher in Columbia than in Stuttgart (overall 167% higher in 2009 and 34% higher in 2010) (Table 2). The range of ureide concentrations among the accessions was also wider in Columbia for both years compared to those measured in Stuttgart (Figure 2A).

**Table 2:** Descriptive statistics of the ureide data (μmol g^−1^) across the 374 accessions evaluated in this study for each of the four environments and the mean across environments

Quartiles	Environment*^a^*				Mean*^b^*
	C09	C10	S09	S10	
Maximum	50.51	43.72	19.56	35.17	33.77
75% Quartile	36.97	28.58	13.58	20.72	24.25
Median	33.07	23.64	12.01	17.60	21.91
25% Quartile	29.33	19.83	10.93	15.27	19.82
Minimum	18.42	10.03	7.42	8.95	13.86
**Summary Statistics**					
Mean	33.07	24.30	12.37	18.17	21.99
SD	6.17	6.33	2.12	3.97	3.32
SEM	0.32	0.33	0.11	0.21	0.17
Upper 95% mean	33.70	24.95	12.59	18.58	22.33
Lower 95% mean	32.44	23.66	12.16	17.77	21.66
N	374	374	374	374	374
Variance	38.04	40.12	4.51	15.80	11.01
Skewness	0.13	0.37	0.61	0.59	0.15
Kurtosis	−0.12	0.05	0.41	0.74	−0.03
CV	18.65	26.06	17.16	21.87	15.09

a C09 and C10 = 2009 and 2010, Columbia, MO, USA; S09 and S10 = 2009 and 2010 Stuttgart, AR, USA.

b Average across the four environments.

By treating accessions (*i.e.*, genotypes) as random effects, ANOVA showed that the variability due to accessions (*i.e.*, heritability) was 33%, 32%, 23%, and 38% for the C09, C10, S09, and S10 environments, respectively. Combined across environments, the total variability was 32% (14% for accessions plus 18% for environment × accession interactions). In all cases, the random effects were significant based on the 95% CIs of the variance component estimates. ANOVA across environments and treating accessions as fixed effects revealed significant differences among accessions (F = 4.24; *P* < 0.0001) and a significant accession × environment interaction (F = 2.17; *P* < 0.0001). Regression of accession means between locations within each year indicated little correspondence (r^2^ = 0.12 and *P* < 0.0001 in 2009 and r^2^ = 0.02 and *P* = 0.0058 in 2010). Better correspondence between years was observed for the Columbia (r^2^ = 0.29, *P* < 0.0001) than the Stuttgart (r^2^ = 0.07, *P* < 0.0001) location.

For each environment, the 374 accessions were ranked from lowest to highest ureide concentration and then the average ranking was generated across all four environments. Table 3 shows the 20 accessions (approximately 5% of the total number of accessions) with the lowest average ranking and the 20 accessions with the highest average ranking along with the ureide concentration in each environment. PI 507424 had the lowest average ranking and PI 424292 had the highest average ranking (Table 3). Overall, the average ranking indicates that these 40 accessions were more consistent in their respective category (high or low ureide concentration) and they likely represent the most consistent extremes for ureide concentration among the 374 accessions evaluated. The 20 accessions with the lowest average rank for ureide concentration across environments were from Japan (seven accessions), China (six), South Korea (four), North Korea (two), and Georgia (one) (Table 3). However, for the 20 accessions with the highest average rank, 16 were from South Korea, three were from Japan, and one was from China (Table 3).

**Table 3:** The 20 accessions with the lowest and highest average ranking for ureide concentration across all four environments

Order	Accession	Country of Origin	Province	Environment*^a^*			
				C09	C10	S09	S10
*Lowest Ureide Concentrations*				μmol g^−1^			
1	PI507424	Japan	Kanto	26.5	18.9	10.1	12.8
2	PI377574	Japan	Iwate	27.2	18.5	10.2	12.9
3	PI532462B	China	Hebei	26.7	19.8	10.0	11.9
4	PI594280B	Japan	Nagano	25.3	17.6	10.6	15.1
5	PI603417	China	Liaoning	25.0	15.3	11.1	14.7
6	PI360846	Japan	Unknown	29.8	17.6	9.5	12.8
7	PI417107	Japan	Tohoku	30.4	14.5	9.8	13.7
8	PI567572B	China	Shandong	25.0	13.5	11.7	13.8
9	PI603911C	North Korea	Unknown	25.4	21.1	10.1	15.0
10	PI612612A	North Korea	Unknown	26.1	21.2	10.6	14.6
11	PI399091	South Korea	Cholla Puk	23.8	19.9	11.3	15.1
12	PI597477	South Korea	Unknown	25.8	17.0	10.8	16.8
13	PI408013	South Korea	Cholla Nam	30.0	17.7	11.5	13.0
14	PI404161	Georgia	Unknown	30.2	19.7	10.9	14.1
15	PI417028	Japan	Kanto	28.0	16.3	11.7	14.2
16	PI567620B	China	Henan	27.2	16.7	10.8	17.0
17	PI567381A	China	Shaanxi	25.6	17.7	10.2	17.6
18	PI430625	China	Unknown	31.3	19.3	10.9	13.6
19	PI507395	Japan	Kanto	31.0	20.5	10.6	14.1
20	PI398283	South Korea	Kyonggi	26.0	19.9	11.0	16.7
			**Average**	27.3	18.1	10.7	14.5
*Highest Ureide Concentrations*							
355	PI567449	China	Shanxi	36.0	26.0	13.6	19.0
356	PI274423	Japan	Miyagi	38.9	24.6	13.9	18.8
357	PI424232A	South Korea	Kyonggi	36.4	33.2	12.2	19.7
358	PI532466A	South Korea	Chungchong Puk	34.6	25.4	13.7	20.4
359	PI398532	South Korea	Chungchong Puk	35.3	28.6	13.0	19.0
360	PI406707	South Korea	Kyonggi	36.2	24.0	13.4	20.5
361	PI424435	South Korea	Cholla Nam	37.8	29.0	12.1	20.2
362	PI508293	South Korea	Cholla Nam	34.5	25.5	13.0	27.4
363	PI424535A	South Korea	Kyongsang Nam	35.5	23.8	14.0	21.2
364	PI507368	Japan	Tohoku	37.4	29.4	12.7	18.9
365	PI408255B	South Korea	Kyongsang Nam	40.3	24.5	13.5	19.7
366	PI424357A	South Korea	Chungchong Nam	38.2	29.7	11.9	22.9
367	PI416942	Japan	Kyushu	36.4	32.5	13.3	18.5
368	PI398730	South Korea	Chungchong Nam	33.6	28.0	13.2	22.0
369	PI424546A	South Korea	Kyongsang Puk	38.6	26.5	12.7	20.9
370	PI408212A	South Korea	Kyongsang Nam	36.1	30.8	13.2	19.5
371	PI398872	South Korea	Kyonggi	36.6	30.9	12.8	20.6
372	PI408318A	South Korea	Kyongsang Nam	36.4	27.3	14.0	20.2
373	PI398319	South Korea	Kyonggi	34.8	27.8	14.1	21.2
374	PI424292	South Korea	Chungchong Nam	39.5	31.7	13.2	21.1
			**Average**	36.7	28.0	13.2	20.6

Ureide concentrations (μmol g^-1^) for each environment are shown.

a C09 and C10 = 2009 and 2010, Columbia, MO USA; S09 and S10 = 2009 and 2010 Stuttgart, AR USA.

### SNP-ureide associations

Potential marker associations with ureide concentration were evaluated by comparing the BLUP mean Log(ureide) concentrations of the two homozygous marker alleles for each of the 33,957 SNP markers with a minor allele frequency ≥0.03 across all 374 accessions. Log values were used to equalize variances among environments and BLUP means were used to help reduce the effect of extreme values (see Figure 2). For each marker, data were analyzed independently within each of the four environments as well as for the overall mean across all four environments. To help control false-positive associations, analyses were conducted with adjustments for population structure (Q-matrix) and genetic relatedness (K-matrix) (Yu *et al.* 2006; Zhu *et al.* 2008). On average the Q- and K-matrix adjustments reduced the number of significant associations detected by approximately 68% at *P* = 0.10 up to approximately 99% at *P* ≤ 0.0001 across environments and for the overall mean (data not shown).

Adjusting for population structure and genetic relatedness and using a stringent probability threshold of *P* ≤ 0.0001 identified 40 SNPs with significant associations with ureide concentration in at least one of the four environments (8, 14, 14, and 4 SNPs for C09, C10, S09, and S10, respectively) as well as 15 significant SNP associations with the overall mean. Of these SNPs, seven were significant both in one environment and for the overall mean. Thus, a total of 48 unique SNPs were identified as significant (*P* ≤ 0.0001) in at least one environment, with the overall mean, or both. A list of these SNPs and their details are provided in Table S1.

The above results considered associations within each individual environment and for the mean ureide concentration across the four environments. To further take advantage of the multiple environments used in this study, we also calculated the joint probability (Mendenhall and Scheaffer 1973) of SNP–trait associations in all two-environment or three-environment combinations and collectively applied an adjustment for multiple testing (Benjamini and Hochberg 1995) threshold of *P* ≤ 0.01. No SNPs in any of the three-environment combinations met the *P* ≤ 0.01 threshold. However, 141 SNPs met the threshold in at least one of the two-environment combinations. A list of these SNPs and their details are provided in Table S1.

**Figure 3:**
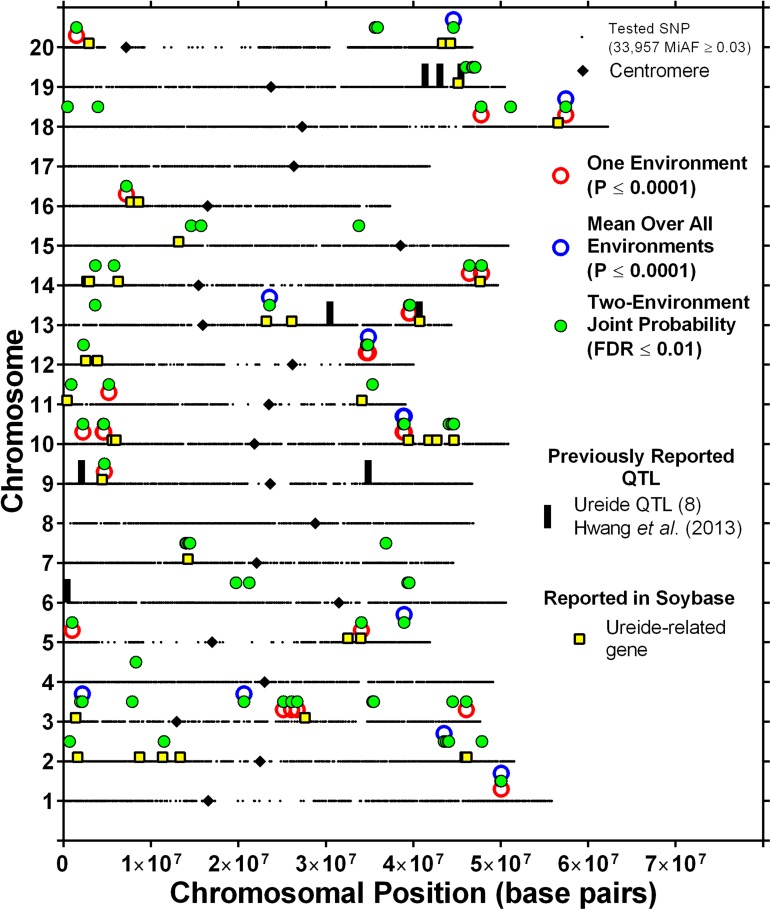
Genomic locations of SNP markers showing significant associations with ureide concentration.

All 15 of the SNPs associated with the mean over all four environments and all but five of the 40 SNPs identified in at least one individual environment were also found to be significantly associated by the joint probability analysis across environments. Thus, a total of 146 (141+5) unique SNPs were identified in individual environments, by the mean overall environments, or by considering two-environment combinations. The relative genomic locations of these 146 SNPs are shown in Figure 3. Considering that closely spaced SNPs likely identify the same locus, these 146 SNPs comprise 53 putative loci (Table 4; Figure 3). Table 4 presents a summary of the SNP information at each putative locus. For those putative loci identified by multiple SNPs, one representative SNP is shown in Table 4, but information for all 146 individual SNPs is shown in Table S1. The number of SNPs tagging each putative locus ranged from 1 to 26 (locus 21, Table 4), with nearly half (24) of the putative loci being identified by multiple SNPs.

**Table 4:** Ureide putative loci identified by at least one of three criteria

Locus	Representative SNP ID*^a^*	CHR*^b^*	Location*^c^*	Number of SNP*^d^*	Range*^e^*	Average MAF*^f^*	Number of Significant SNPs (*P* ≤ 0.0001)		Joint Probability*^i^*	Number of Environments*^j^*	Average Effect*^k^*
							By Environment*^g^*	By Mean*^h^*			
1	ss715580071	1	50,063,918	4	68,465	0.11	1	1	3	4	−7.9%
2	ss715583823	2	708,417	1	0	0.19	0	0	1	2	−4.9%
3	ss715581015	2	11,495,939	2	1069	0.46	0	0	2	3	−7.7%
4	ss715582594	2	43,532,821	6	518,254	0.28	0	1	1	2	6.3%
5	ss715583145	2	47,853,972	2	881	0.08	0	0	1	2	9.1%
6	ss715584759	3	2,153,378	4	262,988	0.08	0	2	4	4	10.6%
7	ss715586962	3	7,856,828	1	0	0.20	0	0	1	2	7.1%
8	ss715584721	3	20,637,413	1	0	0.09	0	1	3	3	10.0%
9	ss715584904	3	25,157,184	1	0	0.05	1	0	2	3	−16.7%
10	ss715584929	3	26,087,333	2	650,448	0.05	2	0	2	3	−16.7%
11	ss715585337	3	35,465,638	3	119,877	0.06	0	0	1	2	−11.8%
12	ss715586306	3	44,514,931	1	0	0.05	0	0	2	3	−17.8%
13	ss715586464	3	46,055,685	5	16,572	0.14	2	0	2	3	−6.9%
14	ss715589403	4	8,293,234	1	0	0.26	0	0	1	2	6.9%
15	ss715592660	5	990,860	1	0	0.04	1	0	2	3	−12.8%
16	ss715590995	5	34,062,898	2	2035	0.18	1	0	1	2	4.6%
17	ss715591699	5	38,950,372	1	0	0.36	0	1	1	2	−6.2%
18	ss715593834	6	19,735,501	1	0	0.03	0	0	1	2	−12.6%
19	ss715593885	6	21,246,909	1	0	0.04	0	0	2	3	−15.1%
20	ss715594357	6	39,539,890	3	180,383	0.22	0	0	3	3	−8.1%
21	ss715596242	7	14,463,247	26	535,597	0.13	0	0	1	2	−13.8%
22	ss715597481	7	36,898,121	3	11,801	0.43	0	0	1	2	6.7%
23	ss715605038	9	4,668,353	1	0	0.42	1	0	0	1	7.4%
24	ss715605875	10	2,222,491	3	8533	0.04	3	0	3	4	18.2%
25	ss715607632	10	4,626,579	5	97,655	0.11	4	0	2	3	−5.9%
26	ss715606803	10	38,989,319	3	157,089	0.04	3	3	4	4	14.3%
27	ss715607408	10	44,087,050	8	561,464	0.11	0	0	1	2	−10.5%
28	ss715611260	11	870,600	1	0	0.04	0	0	1	2	9.9%
29	ss715610836	11	5,189,874	1	0	0.09	1	0	2	3	−8.5%
30	ss715610165	11	35,340,289	1	0	0.08	0	0	1	2	9.6%
31	ss715611822	12	2,293,554	6	23,011	0.07	0	0	1	2	9.3%
32	ss715612491	12	34,883,880	7	264,564	0.25	5	3	4	4	7.9%
33	ss715615937	13	3,640,040	2	13,190	0.26	0	0	1	2	−7.0%
34	ss715614152	13	23,553,775	1	0	0.16	0	1	2	3	9.2%
35	ss715616229	13	39,596,350	9	136,654	0.06	9	0	3	4	17.1%
36	ss715618524	14	3,648,283	1	0	0.29	0	0	1	2	−5.0%
37	ss715619708	14	5,784,050	1	0	0.11	0	0	1	2	8.1%
38	ss715619138	14	46,425,097	1	0	0.05	1	0	1	2	−8.9%
39	ss715619360	14	47,798,437	1	0	0.04	1	0	3	4	−11.3%
40	ss715620804	15	14,589,880	2	24,500	0.08	0	0	1	2	9.7%
41	ss715621024	15	15,742,691	1	0	0.14	0	0	1	2	8.5%
42	ss715621644	15	33,766,611	1	0	0.23	0	0	1	2	−8.8%
43	ss715625421	16	7,185,085	1	0	0.04	1	0	2	3	−22.8%
44	ss715630642	18	458,242	1	0	0.24	0	0	1	2	6.2%
45	ss715630474	18	3,941,529	1	0	0.19	0	0	1	2	8.1%
46	ss715630773	18	47,770,640	1	0	0.25	1	0	0	1	9.4%
47	ss715631152	18	51,128,392	1	0	0.31	0	0	1	2	−5.4%
48	ss715631865	18	57,431,519	3	23,458	0.09	1	1	2	3	8.7%
49	ss715635554	19	46,011,915	1	0	0.22	0	0	1	2	4.9%
50	ss715635623	19	46,822,681	4	315,458	0.25	0	0	2	3	6.7%
51	ss715636704	20	1,489,483	1	0	0.06	1	0	1	2	−8.5%
52	ss715637738	20	35,635,277	3	286,853	0.10	0	0	1	2	−10.2%
53	ss715638654	20	44,601,158	1	0	0.07	0	1	2	3	9.6%

Criteria: (1) significant (*P* ≤ 0.0001) SNP association in at least one of the four environments; (2) significant (*P* ≤ 0.0001) SNP association with the mean across all four environments; and (3) significant (FDR adjusted *P* ≤ 0.01) joint probability SNP association in at least one of all possible two-environment combinations. For loci identified by multiple SNPs, only the SNP with the largest effect is shown.

a NCBI (National Center for Biotechnology Information) submitted SNP ID.

b Glycine max chromosome number.

c Location of the SNP on the chromosome in bp.

d Number of significant SNP associations identifying the putative locus.

e Range in bp over which the SNPs identifying the putative locus were located.

f Minor allele frequency (MAF) averaged over all significant SNP associations for the respective locus.

g Number of significant SNP associations in one or more environments at the *P* ≤ 0.0001 level for the respective locus.

h Number of significant SNP associations with the mean across environments at the *P* ≤ 0.0001 level for the respective locus.

i Number of different two-environment combinations with at least one significant SNP at the respective locus.

j Number of different environments for which at least one of the SNPs tagging the putative locus was significant.

k The average effect was calculated as the percent change in ureide concentration from back-transformed differences in ureide concentrations (major to minor allele) averaged over all significant SNPs tagging a locus.

Of the 53 total putative loci identified, 19 were identified by at least one SNP with a significant association in one or more individual environments (by environment, Table 4; Figure 3). Ten putative loci were identified by one or more SNPs associated with the mean over all environments (by mean, Table 4; Figure 3). Four putative loci (loci 1, 26, 32, and 48, Table 4; Figure 3) were identified by SNPs with significant associations both in individual environments and with the mean over all environments. All but two of the 53 putative loci (loci 23 and 46; Table 4; Figure 3) were identified by at least one two-environment combination in the joint probability analysis. Both of the loci not identified in the joint probability analysis were each identified by a single SNP in one environment (Table 4). Considering both significant associations in individual environments and the joint probability over environments, 16 of the putative loci had three of the four environments contributing a significant SNP association, and for seven putative loci, all four environments contributed (Table 4).

The average effect of all SNPs comprising a given locus is shown as the average percent change in ureide concentration between the mean of those genotypes with the major allele and those with the minor allele (Table 4). Thus, those loci with a negative percent change indicate that the genotypes with the minor allele had a greater ureide concentration than those with the major allele. The average percent change within a putative locus ranged from −22.8 to 18.2%. Twenty-six of the 53 putative loci were associated with an increase in ureide concentration for those genotypes having the minor allele. For those putative loci tagged by multiple SNPs, the response (negative or positive) was the same across all SNPs comprising that locus. Values for each significant SNP are shown in Table S1.

Two of the 53 putative loci identified based on our stringent criteria were near ureide QTL (on chromosomes 13 and 19) previously identified by Hwang *et al.* (2013) in a biparental mapping population (Figure 3). However, application of a lower threshold in a single environment or with the overall mean revealed at least one significant SNP near all eight QTL identified by Hwang *et al.* (2013). This illustrates that more stringent criteria, although providing greater confidence in the identified SNP–ureide associations, may not detect other “real” associations revealed using less stringent criteria. Nonetheless, while the use of more stringent thresholds may eliminate some real SNP–trait associations, we have greater confidence that those SNPs that meet the more stringent thresholds warrant in-depth evaluation.

In addition to reported ureide QTL, a search was conducted in SoyBase for genes that might be related to ureide metabolism and that were located within 3 Mbp of the 53 putative loci shown in Figure 3. This search revealed 38 likely ureide-related genes that are located near 30 of the putative loci identified in this study (Figure 3; Table 5). The genes identified were directly involved in the synthesis of ureides (*i.e.*, uricase), the catabolism of ureides (*i.e.*, allantoate and ureidoglycolate amidohydrolases), or in a biochemical pathway related to ureide metabolism (*i.e.*, nucleotidases, etc.).

**Table 5:** Ureide-related genes identified near the putative loci shown in Figure 3 and Table 4

Gene Model	CHR*^a^*	Start	End	Nearest Putative Locus (Table 4)	Database Reference	Annotation
Glyma.02g018700	2	1,608,638	1,614,322	2	UniRef100_Q8S3J3	Hydroxyisourate hydrolase
Glyma.02g096500	2	8,678,024	8,680,049	3	UniRef100_A9PGT3	Amidohydrolase family protein
Glyma.02g116300	2	11,326,566	11,332,842	3	UniRef100_G7JZ34	Ureide permease
Glyma.02g129800	2	13,316,095	13,324,448	3	UniRef100_B9HIU1	Adenosine deaminase
Glyma.02g276400	2	45,939,610	45,940,658	4	UniRef100_Q8H1P4	Urease accessory protein
Glyma.02g278600	2	46,109,001	46,113,962	5	UniRef100_G7K315	Dihydropyrimidinase
Glyma.03g013600	3	1,379,993	1,390,392	6	UniRef100_G7KQ26	Adenosine deaminase
Glyma.03g093200	3	27,618,965	27,624,224	9/10	UniRef100_I1JMC0	hydroxyisourate hydrolase
Glyma.05g132200	5	32,517,599	32,522,450	16	UniRef100_B9H494	Nucleotidase
Glyma.05g146000	5	33,967,992	33,976,067	16	UniRef100_I1K3K3	Urease
Glyma.07g121800	7	14,214,013	14,214,721	21	UniRef100_K4GMG6	Beta-glucosidase
Glyma.09g050800	9	4,413,615	4,418,223	23	UniRef100_A9GYV1	Allantoate amidohydrolase
Glyma.10g060100	10	5,577,340	5,582,963	25	UniRef100_Q8S4Q4	Nodulin
Glyma.10g060200	10	5,585,676	5,601,516	25	UniRef100_I6WUR4	Glutamine synthetase
Glyma.10g063100	10	5,977,612	5,979,703	25	AT5G03555.1	Allantoin family protein
Glyma.10g160200	10	39,436,452	39,439,106	26	UniRef100_N1R116	Guanine Deaminase
Glyma.10g184900 (Glyma10g32850)*^b^*	10	41,781,111	41,786,726	26	GmUAH1: Werner *et al.* 2013	Ureidoglycolate amidohydrolase
Glyma.10g195000	10	42,702,305	42,705,748	27	UniRef100_G7I312	Nucleotidase
Glyma.10g213900	10	44,627,193	44,629,900	27	AT2G35820.1	Ureidoglycolate hydrolases
Glyma.11g005100	11	393,295	397,851	28	UniRef100_G7ID60	Nucleotidase
Glyma.11g248700	11	34,108,581	34,116,114	30	UniRef100_Q949H4	Urease
Glyma.12g033600	12	2,527,643	2,529,980	31	UniRef100_T2DPS1	Urease accessory protein
Glyma.12g053800	12	3,865,217	3,871,740	31	UniRef100_Q08IT7	Beta-glucosidase
Glyma.13g119100	13	23,184,355	23,191,375	34	UniRef100_G7JR1	Nucleotidase
Glyma.13g147700	13	26,109,258	26,111,357	34	AT5G03555.1	Allantoin family protein
Glyma.13g312100	13	40,721,652	40,729,349	35	UniRef100_G7L867	Adenosine deaminase
Glyma.14g036000	14	2,678,945	2,685,696	36	UniRef100_G7K315	Dihydropyrimidinase
Glyma.14g039400	14	2,958,726	2,960,969	36	UniRef100_Q8H1P4	Urease accessory protein
Glyma.14g074800	14	6,263,339	6,264,169	37	UniRef100_B9IBG2	Nucleotidase
Glyma.14g211700	14	47,655,932	47,660,727	38/39	UniRef100_G7IF65	Beta-glucosidase
Glyma.15g156900 (Glyma15g16870)*^b^*	15	13,142,206	13,147,912	40/41	GmAAH2: Werner *et al.* 2013	Allantoate amidohydrolase
Glyma.16g076200	16	7,722,869	7,723,760	43	UniRef100_G7JTT9	Dihydroorotase
Glyma.16g080700	16	8,575,698	8,583,753	43	UniRef100_I1MM35	hydroxyisourate hydrolase
Glyma.18g284800	18	56,547,660	56,552,282	48	UniRef100_A9PGT3	Amidohydrolase family protein
Glyma.19g193700	19	45,128,512	45,131,581	49/50	UniRef100_G7L609	Ribonucleoside hydrolase
Glyma.20g026300	20	2,894,580	2,905,241	51	UniRef100_G7JHH4	Beta-glucosidase
Glyma.20g195000	20	43,345,158	43,349,435	53	UniRef100_G7I312	Nucleotidase
Glyma.20g205500 (Glyma20g34790)*^b^*	20	44,242,765	44,248,456	53	GmUAH2: Werner *et al.* 2013	Ureidoglycolate amidohydrolase

All genes are from the Glyma2.0 assembly (www.soybase.org).

a *Glycine max* chromosome number.

b Version reported by Werner *et al.* (2013).

## Discussion

Even though a relatively large number of SNPs (33,957 SNPs; MAF ≥3%) were evaluated in this study, gaps of various lengths in the coverage of almost every chromosome (particularly chromosomes 1, 5, 11, 12, and 20) are visible in Figure 3. Many of these gaps are near centromere locations as reported in SoyBase and shown in Figure 3. Chromosomal regions around centromeres have long been known to have less recombination and greater heterochromatic DNA (Slatis 1955; Haupt *et al.* 2001; Westphal and Reuter 2002; Talbert and Henikoff 2010). The lack of SNP variability in these genomic regions is not surprising.

To help control false positives we used BLUP means (to reduce the effect of extreme values) and accounted for both population structure (Q-matrix) and genetic relatedness (K-matrix) (Yu *et al.* 2006; Zhu *et al.* 2008; Dhanapal *et al.* 2015a,b). We also applied high thresholds for reporting significant associations with ureide concentration in each environment or with the overall mean (*P* ≤ 0.0001). Additionally, we examined associations over multiple environments using joint probabilities adjusted for multiple testing (Benjamini and Hochberg 1995) at a threshold of *P* ≤ 0.01. In total, we identified 53 putative loci (Figure 3) associated with ureide concentration. Of these, 29 were identified by a single SNP (Table 4). Lowering the stringency levels in the analysis would likely identify other SNP associations near these loci but may also increase the number of false-positive associations detected. All but two of the 53 putative loci were identified in more than one environment (loci 23 and 46, Table 4). Of those identified in multiple environments, 28 were identified using data from two environments, 16 were identified from three environments, and seven were identified from all four environments. Loci with significant SNP-trait associations over multiple independent environments may indicate that the associated genes are more stably expressed (*i.e.*, less environmental influence). The stringent conditions used in the analysis provides confidence that these loci warrant more detailed investigation.

For 26 of the 53 putative loci, the minor allele was associated with an increase in ureide concentration (negative values in Table 4). Of the five loci with the largest increases in ureide concentration associated with a minor allele, three were near two different putative hydroxyisourate hydrolase genes (Table 5). One was at locus 43 (chromosome 16, −22.8%; Table 4) and the other two were at loci 9 and 10 (both on chromosome 3, Table 4). The two loci on chromosome 3 are near each other and had the same effect on ureide concentration (−16.7%; Table 4). Potentially these loci are not independent. At least one hydroxyisourate hydrolase gene has been shown to play a role in ureide metabolism (Raychaudhuri and Tipton 2002). A more thorough examination of these two putative hydroxyisourate hydrolase genes in the accessions with the minor allele associations may provide previously unknown genetic variation associated with ureide concentrations in soybean. Interestingly, the locus with the second largest increase in ureide concentration (locus 12, −17.8%, Table 4) associated with a minor allele was also on chromosome 3. However, it is likely far enough away from the other putative loci on chromosome 3 to be independent.

Two of the four loci with the largest increases associated with elevated ureide concentration for the major allele were located on chromosome 10 (loci 24 and 26; 18.2% and 14.3%, respectively, Table 4 and Figure 3). Both loci were tagged by three SNPs each but significant associations were detected in all four environments (Table 4). No putative ureide-related gene was identified near locus 24 and an ureidoglycolate amidohyrolase (discussed below) was near the other. The other two loci with the largest effect (loci 6 and 35; Table 4) were located near different putative adenosine deaminase genes (Table 5). Interestingly, one of these (locus 35, Table 4; and see chromosome 13 in Figure 3) was also near one of the putative QTL identified by Hwang *et al.* (2013). Both of these loci were tagged by multiple SNPs (four and nine SNPs, respectively, Table 4) and significant associations were detected in all four environments (Table 4). Adenosine deaminases are involved in purine metabolism; however, their full role in plants is not well understood. In fact, Dancer *et al.* (1997) concluded that plants do not contain adenosine deaminase, although others have reported low levels (Edwards 1996). The large effect of these two putative loci on ureide concentration and their location near putative adenosine deaminase genes may provide a path for research to investigate and more fully understand the role of adenosine deaminases in plants.

Werner *et al.* (2013) examined two gene copies of allantoate amidohydrolase (GmAAH1 and GmAAH2), ureidoglycine aminohydrolase (GmUGlyAH1 and GmUGlyAH2), and ureidoglycolate amidohydrolase (GmUAH1 and GmUAH2), which are all involved in ureide hydrolysis (Werner *et al.* 2013). Three of these genes, GmUAH1 (Glyma10g32850, chromosome 10), GmAAH2 (Glyma15g16870, chromosome 15), and GmUAH2 (Glyma20g34790, chromosome 20), were each near a different locus of the 53 putative loci (see Table 5 and Figure 3). For GmUAH2 (chromosome 20), one nearby significant SNP association was detected (Table 4, locus 53), while three (Table 4, locus 26) and two (Table 4, locus 40) nearby significant SNP associations marked the loci near GmUAH1 (chromosome 10) and GmAAH2 (chromosome 15), respectively. For all three loci, the major allele was associated with an increase in ureide concentration (Table 4) and, thus, the minor allele was associated with a decrease in ureide concentration. Potentially, the reduced ureide concentration in the accessions with the minor alleles near these genes might be associated with more active/efficient ureide metabolism. Reduced petiole ureide concentrations have been associated with increased drought tolerant N-fixation, possibly through the elimination/reduction of feedback inhibition caused by a buildup of ureides (Sinclair and Serraj 1995; Serraj and Sinclair 1996; de Silva *et al.* 1996; Gordon *et al.* 1997; Purcell *et al.* 1998; Serraj *et al.* 1999, 2001; Vadez *et al.* 2000; King and Purcell 2005; Ladrera *et al.* 2007). Thus, if these three loci are associated with greater ureide catabolism as a result of more active/efficient alleles of these hydrolases, then the accessions with the minor allele may also exhibit greater drought-tolerant N-fixation. While no SNPs evaluated in this study were located between the start and stop positions of GmUAH1, GmAAH2, and GmUAH2, comparisons of the sequences of these genes between genotypes with the major and minor alleles for the nearby significant SNP could be of interest. Additionally, confirmation of tolerance and more detailed investigation of these accessions may aid in the identification of the molecular mechanisms associated with drought-tolerant N-fixation.

Duran and Todd (2012) identified four allantoinase (E.C. 3.5.2.5) genes that they designated GmALN1, GmALN2, GmALN3, and GmALN4 (corresponding to Glyma15g07910, Glyma13g31430, Glyma15g07920, and Glyma13g31420). GmALN1 and GmALN3 are on chromosome 15 and GmALN2 and GmALN4 are on chromosome 13. On both chromosomes, the two respective genes are very closely spaced (within approximately 11,000 bp). None of the 53 putative loci identified using the stringent conditions were near the location of these genes on either chromosome. However, at lower stringency (*P* ≤ 0.01), significant SNP associations were detected within 0.4 MB (chromosome 13) and 0.6 MB (chromosome 15) for both pairs of genes. Thus, the identification of these SNPs associated with well-characterized ureide-related genes again indicates that very stringent criteria may mask true associations. This emphasizes the necessity for approaches that balance the need to identify true associations with the need to eliminate false positives, because at lower stringencies many more likely false-positive SNP associations can be identified. Without independent information (*i.e.*, Duran and Todd 2012; Hwang *et al.* 2013) or further confirmation, false positives at lower stringencies are especially problematic.

Other putative loci (Figure 3) were not near any gene annotated as ureide related in SoyBase. This may represent a lack of knowledge about the function of genes in the region or, even with the high stringencies used, some of the putative loci may represent false positives. Nonetheless, even though many of the other loci are corroborated by previously identified QTL or annotated genes, these loci also warrant further investigation.

In this study, 53 putative loci associated with ureide concentration were identified. Two of the putative loci were located near previously reported QTL associated with ureide concentration and 30 loci were located near genes associated with ureide metabolism. Potentially, these results indicate variation in known genes that require further investigation. The remaining loci may represent new genes affecting ureide concentration (biosynthesis, transport, degradation, etc.) and also warrant further in-depth investigation. Confirmation of these loci could be accomplished through quantifying segregation in appropriately constructed biparental mapping populations. Further investigations such as expression analyses and sequencing of important known genes (*i.e.*, specific uricase and amidohydrolase genes) near the loci identified in the accessions comprising the minor SNP frequency may reveal novel insights into the regulation of ureide synthesis or catabolism. Ultimately, confirmation of the putative loci identified in this study will provide new sources of variation for use in breeding programs developing improved soybean cultivars.

## 

## References

[bib1] Benjamini Y. Hochberg Y., 1995 Controlling the false discovery rate: A practical and powerful approach to multiple testing. J. R. Stat. Soc., B 57: 289–300.

[bib2] Coleto I. Pineda M. Rodin A. P. De Ron A. M. Alamillo J. M., 2014 Comparison of inhibition of N_2_ fixation and ureide accumulation under water deficit in four common bean genotypes of contrasting drought tolerance. Ann. Bot. (Lond.) 113: 1071–1082. 10.1093/aob/mcu029 PMC3997645 24638821

[bib3] Dancer J. E. Hughes R. G. Lindell S. D., 1997 Adenosine-5′-phosphate demainase. A novel herbicide target. Plant Physiol. 114: 119–129. 9159944 10.1104/pp.114.1.119 PMC158285

[bib4] de Silva M. Purcell L. C. King C. A., 1996 Soybean petiole ureide response to water deficits and decreased transpiration. Crop Sci. 36: 611–616.

[bib5] Deshmukh R. Sonah H. Patil G. Chen W. Prince S., 2014 Integrating omic approaches for abiotic stress tolerance in soybean. Front. Plant Sci. 5: 244 . 10.3389/fpls.2014.00244 24917870 PMC4042060

[bib6] Dhanapal A. P. Ray J. D. Singh S. K. Hoyos-Villegas V. Smith J. R., 2015 a Genome-wide association study (GWAS) of carbon isotope ratio (δ^13^C) in diverse soybean [*Glycine max* (L.) Merr.] genotypes. Theor. Appl. Genet. 128: 73–91. 25367378 10.1007/s00122-014-2413-9

[bib7] Dhanapal A. P. Ray J. D. Singh S. K. Hoyos-Villegas V. Smith J. R. Purcell L. C. King C. A. Fritschi F. B., 2015 b Genome-wide association analysis of diverse soybean genotypes reveals novel markers for nitrogen traits. Plant Genome DOI: 10.3835/plantgenome2014.11.0086 10.3835/plantgenome2014.11.0086 33228264

[bib8] Duran V. A. Todd C. D., 2012 Four allantoinase genes are expressed in nitrogen-fixing soybean. Plant Physiol. Biochem. 54: 149–155. 22476036 10.1016/j.plaphy.2012.03.002

[bib9] Edwards R., 1996 S-adenosyl-L-methionine metabolism in alfalfa cell cultures following treatment with fungal elicitors. Phytochemistry 43: 1163–1169.

[bib10] Fehr W. R. Caviness C. E. Burmood D. T. Pennington J. S., 1971 Stage of development descriptions for soybeans, *Glycine max* (L.). Merrill. Crop Sci. 11: 929–931.

[bib11] Gordon A. J. Minchin F. R. Skot L. James C. L., 1997 Stress-induced declines in soybean N_2_ fixation are related to nodule sucrose synthase activity. Plant Physiol. 114: 937–946. 12223754 10.1104/pp.114.3.937 PMC158382

[bib12] Hao D., H. Cheng H, Z. Yin, S. Cui, D. Zhang, H. Wang, and D. Yu, 2012 Identification of single nucleotide polymorphisms and haplotypes associated with yield and yield components in soybean (*Glycine max*) landraces across multiple environments. Theor. Appl. Genet. 124: 447–458. 21997761 10.1007/s00122-011-1719-0

[bib13] Haupt W. Fischer T. C. Winderl S. Fransz P. Torres-Ruiz R. A., 2001 The centromere1 (CEN1) region of *Arabidopsis thaliana*: Architecture and functional impact of chromatin. Plant J. 27: 285–296. 11532174 10.1046/j.1365-313x.2001.01087.x

[bib14] Hwang S. King C. A. Davies M. K. Ray J. D. Cregan P. B., 2013 QTL Analysis of shoot ureide and nitrogen concentrations in soybean (*Glycine max* L.[Merr.]). Crop Sci. 53: 2421–2433.

[bib15] Hwang E. Y. Song Q. Jia G. Specht J. E. Hyten D. L., 2014 A genome-wide association of seed protein and oil content in soybean. BMC Genomics 15: 1–12. 24382143 10.1186/1471-2164-15-1 PMC3890527

[bib16] King C. A. Purcell L. C., 2005 Inhibition of N_2_ fixation in soybean is associated with elevated ureides and amino acids. Plant Physiol. 137: 1389–1396. 15778462 10.1104/pp.104.056317 PMC1088329

[bib17] King C. A. Purcell L. C. Bolton A. Specht J. E., 2014 A possible relationship between shoot N concentration and the sensitivity of N_2_ fixation to drought in soybean. Crop Sci. 54: 746–756.

[bib18] Kruskal, J. B., and M. Wish, 1978 *Multidimensional Scaling*, *Sage University Paper Series on Quantitative Applications in the Social Sciences*, *07–011*. Beverly Hills and London: Sage Publications.

[bib19] Ladrera R. Marino D. Larrainzar E. Gonzalez E. M. Arrese-Igor C., 2007 Reduced carbon availability to bacteroids and elevated ureides in nodules, but not in shoots, are involved in the nitrogen fixation response to early drought in soybean. Plant Physiol. 145: 539–546. 17720761 10.1104/pp.107.102491 PMC2048725

[bib20] Mamidi S. Lee R. K. Goos J. R. McClean P. E., 2014 Genome-wide association studies identifies seven major regions responsible for iron deficiency chlorosis in soybean (*Glycine max*). PLoS One 9: e107469. 25225893 10.1371/journal.pone.0107469 PMC4166409

[bib21] Mendenhall W. Scheaffer R. L., 1973 pp. 17–66 in Mathematical Statistics with Applications, Wadsworth Publishing Company, Inc., Belmont, CA.

[bib22] Purcell L. C. Serraj R. de Silva M. Sinclair T. R. Bona S., 1998 Ureide concentration of field-grown soybean in response to drought and the relationship to nitrogen fixation. J. Plant Nutr. 21: 949–966.

[bib23] Purcell S. Neale B. Todd-Brown K. Thomas L. Ferreira M. A., 2007 PLINK: a tool set for whole-genome association and population-based linkage analyses. Am. J. Hum. Genet. 81: 559–575. 17701901 10.1086/519795 PMC1950838

[bib24] Raychaudhuri A. Tipton P. A., 2002 Cloning and expression of the gene for soybean hydroxyisourate hydrolase. Localization and implications for function and mechanism. Plant Physiol. 130: 2061–2068. 12481089 10.1104/pp.011049 PMC166717

[bib25] SAS Institute Inc, 2008 Chapter 53: The MDS procedure, pp. 3698–3735 in *SAS/STAT 9.2 User’s Guide*. SAS Institute Inc., Cary, NC.

[bib26] Serraj R. Sinclair T. R., 1996 Processes contributing to N2-fixation insensitivity to drought in the soybean cultivar Jackson. Crop Sci. 36: 961–968.

[bib27] Serraj R. Vadez V. Denison R. F. Sinclair T. R., 1999 Involvement of ureides in nitrogen fixation inhibition in soybean. Plant Physiol. 119: 289–296. 9880371 10.1104/pp.119.1.289 PMC32231

[bib28] Serraj R. Vadez V. Sinclair T. R., 2001 Feedback regulation of symbiotic N_2_ fixation under drought stress. Agronomie 21: 621–626.

[bib29] Sinclair T. R. Serraj R., 1995 Legume nitrogen fixation and drought. Nature 378: 344.

[bib30] Sinclair T. R. Purcell L. C. Vadez V. Serraj R. Andy King C., 2000 Identification of soybean genotypes with N fixation tolerance to water deficits. Crop Sci. 40: 1803–1809.

[bib31] Slatis H. M., 1955 A reconsideration of the brown-dominant position effect. Genetics 40: 246–251. 17247549 10.1093/genetics/40.2.246 PMC1209718

[bib32] Sonah H. O’Donoughue L. Cober E. Rajcan I. Belzile F., 2014 Identification of loci governing eight agronomic traits using a GBS-GWAS approach and validation by QTL mapping in soya bean. Plant Biotechnol. J. 10.1111/pbi.12249 25213593

[bib33] Song Q. Hyten D. L. Jia G. Quigley C. V. Fickus E. W., 2013 Development and evaluation of SoySNP50K, a high-density genotyping array for soybean. PLoS One 8: e54985. 23372807 10.1371/journal.pone.0054985 PMC3555945

[bib34] Talbert P. B. Henikoff S., 2010 Centromeres convert but don’t cross. PLoS Biol. 8: e1000326 . 10.1371/journal.pbio.1000326 20231873 PMC2834710

[bib35] Vadez V. Sinclair T. R. Serraj R., 2000 Asparagine and ureide accumulation in nodules and shoots as feedback inhibitors of N_2_ fixation in soybean. Physiol. Plant. 110: 215–223.

[bib36] Watanabe S. Matsumoto M. Hakomori Y. Takagi H. Shimada H., 2014 The purine metabolite allantoin enhances abiotic stress tolerance through synergistic activation of abscisic acid metabolism. Plant Cell Environ. 37: 1022–1036. 24182190 10.1111/pce.12218

[bib37] Wen Z. Tan R. Yuan J. Bales C. Du W., 2014 Genome-wide association mapping of quantitative resistance to sudden death syndrome in soybean. BMC Genomics 15: 809. 25249039 10.1186/1471-2164-15-809 PMC4189206

[bib38] Werner A. K. Medina-Escobar N. Zulawski M. Sparkes I. A. Cao F. Q., 2013 The ureide-degrading reactions of purine ring catabolism employ three amidohydrolases and one aminohydrolase in Arabidopsis, soybean, and rice. Plant Physiol. 163: 672–681. 23940254 10.1104/pp.113.224261 PMC3793049

[bib39] Westphal T. Reuter G., 2002 Recombinogenic effects of suppressors of position-effect variegation in *Drosophila*. Genetics 160: 609–621. 11861565 10.1093/genetics/160.2.609 PMC1461983

[bib40] Young E. G. Conway C. F., 1942 On the estimation of allantoin by the Rimini-Schryver reaction. J. Biol. Chem. 142: 839–853.

[bib41] Yu J. Pressoir G. Briggs W. H. Vroh Bi I. Yamasaki M., 2006 A unified mixed-model method for association mapping that accounts for multiple levels of relatedness. Nat. Genet. 38: 203–208. 16380716 10.1038/ng1702

[bib42] Zhang D. Song H. Cheng H. Hao D. Wang H., 2014 The acid phosphatase-encoding gene GmACP1 contributes to soybean tolerance to low-phosphorus stress. PLoS Genet. 10: e1004061 . 10.1371/journal.pgen.1004061 24391523 PMC3879153

[bib43] Zhang J. Song Q. Cregan P. B. Nelson R. L. Wang X., 2015 Genome-wide association study for flowering time, maturity dates and plant height in early maturing soybean (Glycine max) germplasm. BMC Genomics 16: 217. 25887991 10.1186/s12864-015-1441-4 PMC4449526

[bib44] Zhu C. Gore M. Buckler E. S. Jianming Y., 2008 Status and prospects of association mapping in plants. Plant Genome 1: 5–20.

